# *N*-Ethyl-2-[1-(2-hy­droxy-6-meth­oxy­phenyl)ethyl­idene]hydrazinecarbothio­amide

**DOI:** 10.1107/S1600536812039323

**Published:** 2012-09-22

**Authors:** Brian J. Anderson, Christopher J. Kennedy, Jerry P. Jasinski

**Affiliations:** a Department of Chemistry, Keene State College, 229 Main Street, Keene, NH 03435-2001, USA

## Abstract

In the title compound, C_12_H_17_N_3_O_2_S, the dihedral angle between the mean planes of the hydrazinecarbothio­amide group and the benzene ring is 86.8 (4)°. In the crystal, inter­molecular O—H⋯S hydrogen bonds link the mol­ecules into chains along [001]. The crystal studied was an inversion twin, the refined ratio of the twin components being 0.98021 (3):0.01978 (7).

## Related literature

For thio­semicarbazone structures and their biological activity, see: Lobana *et al.* (2009 ▶). For thio­semicarbazones as ligands for metal-catalyzed reactions or hydrogenations, see: Xie *et al.* (2010 ▶); Pelagatti *et al.* (1998 ▶). For reference bond-length data, see: Allen *et al.* (1987 ▶).
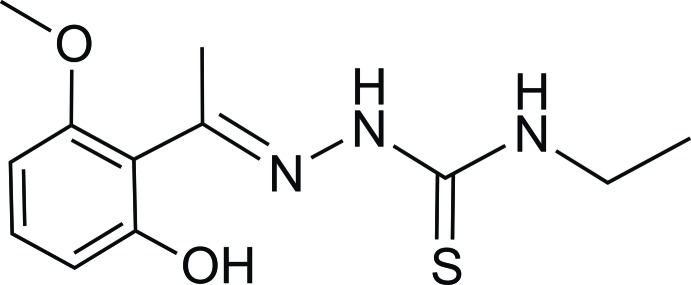


## Experimental

### 

#### Crystal data

C_12_H_17_N_3_O_2_S
*M* *_r_* = 267.35
Monoclinic, 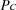

*a* = 8.5681 (6) Å
*b* = 8.0393 (5) Å
*c* = 10.3808 (8) Å
β = 103.510 (7)°
*V* = 695.26 (8) Å^3^
*Z* = 2
Mo *K*α radiation
μ = 0.23 mm^−1^
*T* = 173 K
0.46 × 0.32 × 0.24 mm

#### Data collection

Oxford Xcalibur (Eos, Gemini) diffractometer
Absorption correction: multi-scan (*CrysAlis RED*; Oxford Diffraction, 2010 ▶) *T*_min_ = 0.974, *T*_max_ = 1.000
7338 measured reflections
3987 independent reflections
3287 reflections with *I* > 2σ(*I*)
*R*_int_ = 0.030

#### Refinement

*R*[*F* ^2^ > 2σ(*F* ^2^)] = 0.047
*wR*(*F* ^2^) = 0.128
*S* = 1.05
3987 reflections
173 parameters
2 restraints
H atoms treated by a mixture of independent and constrained refinement
Δρ_max_ = 0.63 e Å^−3^
Δρ_min_ = −0.21 e Å^−3^
Absolute structure: Flack (1983 ▶), with 1510 Friedel pairs
Flack parameter: 0.00 (8)

### 

Data collection: *CrysAlis PRO* (Oxford Diffraction, 2010 ▶); cell refinement: *CrysAlis PRO*; data reduction: *CrysAlis RED* (Oxford Diffraction, 2010 ▶); program(s) used to solve structure: *SHELXS97* (Sheldrick, 2008 ▶); program(s) used to refine structure: *SHELXL97* (Sheldrick, 2008 ▶); molecular graphics: *SHELXTL* (Sheldrick, 2008 ▶); software used to prepare material for publication: *SHELXTL*.

## Supplementary Material

Crystal structure: contains datablock(s) global, I. DOI: 10.1107/S1600536812039323/cv5339sup1.cif

Structure factors: contains datablock(s) I. DOI: 10.1107/S1600536812039323/cv5339Isup2.hkl

Supplementary material file. DOI: 10.1107/S1600536812039323/cv5339Isup3.cml

Additional supplementary materials:  crystallographic information; 3D view; checkCIF report

## Figures and Tables

**Table 1:** Hydrogen-bond geometry (Å, °)

*D*—H⋯*A*	*D*—H	H⋯*A*	*D*⋯*A*	*D*—H⋯*A*
O1—H1⋯S1^i^	0.82	2.35	3.1655 (19)	175

Symmetry code: (i) 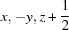.

**Fig. 1.**
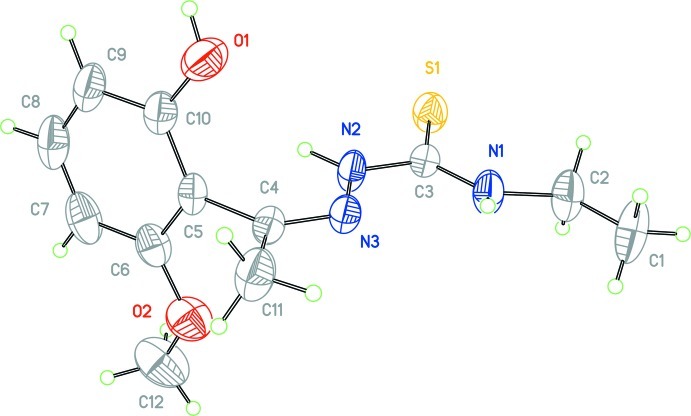
The molecular structure of the title compound with the atom numbering scheme. Displacement ellipsoids are drawn at the 50% probability level. H atoms are presented as small spheres of arbitrary radius.

**Fig. 2.**
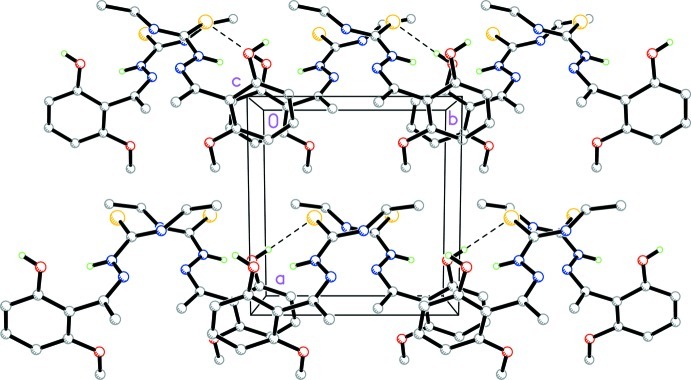
Packing diagram viewed along the c axis. Weak O—H···S intermolecular interactions are shown by dashed lines. C-bound H atoms were omitted for clarity.

## supplementary crystallographic information

### Comment

Thiosemicarbazones are an important class of ligands whose metal complex structures and biological activity have been extensively investigated (Lobana *et al.*, 2009). Recently, thiosemicarbazones have been studied as ligands for metal catalyzed reactions such as Mizoroki–Heck couplings (Xie *et al.*, 2010) and hydrogenations (Pelagatti *et al.*, 1998). The crystal structure of a novel thiosemicarbazone molecule is reported here.

In the title compound, C_12_H_17_N_3_O_2_S (Fig. 1), the dihedral angle between the mean plane of the hydrazinecarbothioamide group (N1/S1/C3/N2/N3) and benzene ring is 86.8 (4)°. Bond lengths are in normal ranges (Allen *et al.*, 1987). In the crystal, the intermolecular O—H···S hydrogen bonds (Table 1) link the molecules into chains in [001] (Fig. 2).

### Experimental

A 50 ml round-bottomed flask was charged with 0.507 g (3.05 mmol) of 2'-hydroxy-6'-methoxyacetophenone and 0.363 g (3.05 mmol) of 4-ethyl-3-thiosemicarbazide followed by 35 ml of methanol, resulting in a clear yellow solution. The solution was refluxed for 5 h, and then the solvent was removed by rotary evaporation. The product was dissolved into 40°C acetonitrile and slowly allowed to cool to 0°C. Translucent crystals were observed after 48 h. (m.p. 458–460 K).

### Refinement

Atoms H1A and H2 were located on a difference map and refined isotropically. The remaining H atoms were placed in their calculated positions and then refined using the riding model, with C—H lengths of 0.93 Å (CH), 0.97 Å (CH_2_) or 0.96 Å (CH_3_) and the O—H length of 0.82 Å. The isotropic displacement parameters for these atoms were set to 1.2 (CH, CH_2_) or 1.5 (CH_3_, OH) times *U*_eq_ of the parent atom. The structure was refined as an inversion twin, with the twin law -1 0 0 0 -1 0 0 0 -1 2 and the refined ratio of twin components being 0.98021 (3):0.01978 (7).

### Crystal data

**Table:**

C_12_H_17_N_3_O_2_S	*F*(000) = 284
*M**_r_* = 267.35	*D*_x_ = 1.277 Mg m^−^^3^
Monoclinic, *P**c*	Mo *K*α radiation, λ = 0.71070 Å
Hall symbol: P -2yc	Cell parameters from 2153 reflections
*a* = 8.5681 (6) Å	θ = 3.2–32.3°
*b* = 8.0393 (5) Å	µ = 0.23 mm^−^^1^
*c* = 10.3808 (8) Å	*T* = 173 K
β = 103.510 (7)°	Chunk, colourless
*V* = 695.26 (8) Å^3^	0.46 × 0.32 × 0.24 mm
*Z* = 2	

### Data collection

**Table:**

Oxford Xcalibur (Eos, Gemini) diffractometer	3987 independent reflections
Radiation source: Enhance (Mo) X-ray Source	3287 reflections with *I* > 2σ(*I*)
Graphite monochromator	*R*_int_ = 0.030
Detector resolution: 16.1500 pixels mm^-1^	θ_max_ = 32.3°, θ_min_ = 3.2°
ω scans	*h* = −12→11
Absorption correction: multi-scan (*CrysAlis RED*; Oxford Diffraction, 2010)	*k* = −11→11
*T*_min_ = 0.974, *T*_max_ = 1.000	*l* = −15→15
7338 measured reflections	

### Refinement

**Table:**

Refinement on *F*^2^	Secondary atom site location: difference Fourier map
Least-squares matrix: full	Hydrogen site location: inferred from neighbouring sites
*R*[*F*^2^ > 2σ(*F*^2^)] = 0.047	H atoms treated by a mixture of independent and constrained refinement
*wR*(*F*^2^) = 0.128	*w* = 1/[σ^2^(*F*_o_^2^) + (0.0636*P*)^2^ + 0.0627*P*] where *P* = (*F*_o_^2^ + 2*F*_c_^2^)/3
*S* = 1.05	(Δ/σ)_max_ < 0.001
3987 reflections	Δρ_max_ = 0.63 e Å^−^^3^
173 parameters	Δρ_min_ = −0.21 e Å^−^^3^
2 restraints	Absolute structure: Flack (1983), with 1510 Friedel pairs
Primary atom site location: structure-invariant direct methods	Flack parameter: 0.00 (8)

### Special details

**Table:**

Geometry. All esds (except the esd in the dihedral angle between two l.s. planes) are estimated using the full covariance matrix. The cell esds are taken into account individually in the estimation of esds in distances, angles and torsion angles; correlations between esds in cell parameters are only used when they are defined by crystal symmetry. An approximate (isotropic) treatment of cell esds is used for estimating esds involving l.s. planes.
Refinement. Refinement of *F*^2^ against ALL reflections. The weighted *R*-factor *wR* and goodness of fit *S* are based on *F*^2^, conventional *R*-factors *R* are based on *F*, with *F* set to zero for negative *F*^2^. The threshold expression of *F*^2^ > σ(*F*^2^) is used only for calculating *R*-factors(gt) *etc*. and is not relevant to the choice of reflections for refinement. *R*-factors based on *F*^2^ are statistically about twice as large as those based on *F*, and *R*-factors based on ALL data will be even larger.

### Fractional atomic coordinates and isotropic or equivalent isotropic displacement parameters (Å^2^)

**Table:**

	*x*	*y*	*z*	*U*_iso_*/*U*_eq_	
S1	0.55727 (6)	0.29581 (7)	0.59523 (6)	0.04628 (16)	
O1	0.7690 (2)	0.0256 (2)	1.0893 (2)	0.0585 (5)	
H1	0.7155	−0.0595	1.0865	0.088*	
O2	1.1860 (2)	0.2488 (3)	0.92524 (19)	0.0549 (5)	
N1	0.6144 (3)	0.5442 (3)	0.7677 (2)	0.0457 (5)	
H1A	0.637 (4)	0.575 (4)	0.839 (3)	0.050 (9)*	
N2	0.7546 (2)	0.3070 (2)	0.83163 (19)	0.0379 (4)	
H2	0.782 (3)	0.198 (4)	0.823 (3)	0.047 (8)*	
N3	0.8239 (2)	0.3855 (2)	0.94956 (18)	0.0344 (4)	
C1	0.4947 (5)	0.8165 (4)	0.7392 (3)	0.0657 (9)	
H1B	0.4404	0.7948	0.8086	0.098*	
H1C	0.4323	0.8925	0.6764	0.098*	
H1D	0.5980	0.8644	0.7765	0.098*	
C2	0.5155 (4)	0.6584 (4)	0.6716 (3)	0.0568 (7)	
H2A	0.4116	0.6086	0.6348	0.068*	
H2B	0.5674	0.6800	0.5996	0.068*	
C3	0.6451 (2)	0.3906 (2)	0.7390 (2)	0.0346 (4)	
C4	0.9297 (2)	0.3025 (2)	1.0323 (2)	0.0333 (4)	
C5	0.9822 (2)	0.1311 (3)	1.0085 (2)	0.0345 (4)	
C6	1.1173 (3)	0.1064 (3)	0.9568 (2)	0.0415 (5)	
C7	1.1719 (3)	−0.0556 (4)	0.9435 (3)	0.0523 (6)	
H7	1.2623	−0.0731	0.9102	0.063*	
C8	1.0913 (4)	−0.1879 (3)	0.9799 (3)	0.0532 (7)	
H8	1.1301	−0.2948	0.9731	0.064*	
C9	0.9556 (3)	−0.1677 (3)	1.0258 (3)	0.0494 (6)	
H9	0.9002	−0.2595	1.0464	0.059*	
C10	0.9014 (3)	−0.0067 (3)	1.0412 (2)	0.0411 (5)	
C11	1.0021 (3)	0.3811 (3)	1.1618 (3)	0.0510 (6)	
H11A	0.9585	0.4908	1.1644	0.077*	
H11B	1.1163	0.3883	1.1730	0.077*	
H11C	0.9782	0.3151	1.2319	0.077*	
C12	1.3067 (4)	0.2315 (5)	0.8506 (3)	0.0717 (9)	
H12A	1.3976	0.1733	0.9029	0.107*	
H12B	1.3397	0.3398	0.8281	0.107*	
H12C	1.2636	0.1700	0.7710	0.107*	

### Atomic displacement parameters (Å^2^)

**Table:**

	*U*^11^	*U*^22^	*U*^33^	*U*^12^	*U*^13^	*U*^23^
S1	0.0464 (3)	0.0416 (3)	0.0444 (3)	0.0038 (3)	−0.0023 (2)	−0.0014 (3)
O1	0.0607 (11)	0.0363 (9)	0.0872 (14)	0.0008 (8)	0.0352 (11)	0.0062 (10)
O2	0.0481 (10)	0.0661 (12)	0.0562 (12)	0.0008 (9)	0.0238 (9)	0.0002 (9)
N1	0.0463 (10)	0.0360 (10)	0.0482 (12)	0.0125 (8)	−0.0027 (9)	0.0005 (9)
N2	0.0434 (9)	0.0266 (8)	0.0390 (10)	0.0100 (7)	0.0001 (8)	0.0012 (7)
N3	0.0367 (8)	0.0284 (8)	0.0371 (9)	0.0056 (7)	0.0066 (7)	0.0024 (7)
C1	0.100 (3)	0.0467 (15)	0.0512 (15)	0.0374 (16)	0.0185 (16)	0.0129 (12)
C2	0.0634 (16)	0.0465 (14)	0.0556 (16)	0.0212 (12)	0.0042 (13)	0.0089 (12)
C3	0.0312 (9)	0.0284 (9)	0.0433 (11)	0.0026 (8)	0.0066 (8)	0.0047 (8)
C4	0.0366 (10)	0.0291 (9)	0.0344 (10)	0.0049 (7)	0.0089 (8)	0.0048 (8)
C5	0.0378 (10)	0.0314 (10)	0.0319 (9)	0.0105 (8)	0.0031 (8)	0.0030 (8)
C6	0.0382 (10)	0.0493 (13)	0.0344 (10)	0.0081 (9)	0.0034 (9)	−0.0021 (9)
C7	0.0464 (12)	0.0651 (17)	0.0438 (12)	0.0242 (12)	0.0074 (10)	−0.0085 (12)
C8	0.0653 (16)	0.0414 (13)	0.0442 (13)	0.0226 (12)	−0.0046 (12)	−0.0054 (10)
C9	0.0603 (15)	0.0317 (11)	0.0504 (14)	0.0123 (10)	0.0012 (12)	0.0045 (10)
C10	0.0426 (11)	0.0355 (10)	0.0432 (12)	0.0084 (9)	0.0062 (9)	0.0030 (9)
C11	0.0660 (15)	0.0382 (12)	0.0429 (13)	0.0100 (11)	0.0007 (11)	−0.0026 (10)
C12	0.0528 (16)	0.114 (3)	0.0560 (17)	−0.0008 (17)	0.0279 (14)	−0.0007 (18)

### Geometric parameters (Å, º)

**Table:**

S1—C3	1.688 (2)	C4—C11	1.484 (3)
O1—C10	1.367 (3)	C4—C5	1.487 (3)
O1—H1	0.8200	C5—C10	1.389 (3)
O2—C6	1.362 (3)	C5—C6	1.399 (3)
O2—C12	1.436 (3)	C6—C7	1.402 (4)
N1—C3	1.311 (3)	C7—C8	1.368 (4)
N1—C2	1.471 (3)	C7—H7	0.9300
N1—H1A	0.76 (3)	C8—C9	1.365 (4)
N2—C3	1.355 (3)	C8—H8	0.9300
N2—N3	1.382 (2)	C9—C10	1.396 (3)
N2—H2	0.92 (3)	C9—H9	0.9300
N3—C4	1.281 (3)	C11—H11A	0.9600
C1—C2	1.482 (4)	C11—H11B	0.9600
C1—H1B	0.9600	C11—H11C	0.9600
C1—H1C	0.9600	C12—H12A	0.9600
C1—H1D	0.9600	C12—H12B	0.9600
C2—H2A	0.9700	C12—H12C	0.9600
C2—H2B	0.9700		
			
C10—O1—H1	109.5	C6—C5—C4	120.3 (2)
C6—O2—C12	117.1 (3)	O2—C6—C5	114.6 (2)
C3—N1—C2	123.2 (2)	O2—C6—C7	125.7 (2)
C3—N1—H1A	121 (2)	C5—C6—C7	119.7 (2)
C2—N1—H1A	116 (2)	C8—C7—C6	119.6 (2)
C3—N2—N3	119.04 (16)	C8—C7—H7	120.2
C3—N2—H2	124.0 (18)	C6—C7—H7	120.2
N3—N2—H2	116.8 (18)	C9—C8—C7	122.0 (2)
C4—N3—N2	116.48 (17)	C9—C8—H8	119.0
C2—C1—H1B	109.5	C7—C8—H8	119.0
C2—C1—H1C	109.5	C8—C9—C10	118.9 (3)
H1B—C1—H1C	109.5	C8—C9—H9	120.5
C2—C1—H1D	109.5	C10—C9—H9	120.5
H1B—C1—H1D	109.5	O1—C10—C5	116.17 (19)
H1C—C1—H1D	109.5	O1—C10—C9	123.0 (2)
N1—C2—C1	109.1 (2)	C5—C10—C9	120.8 (2)
N1—C2—H2A	109.9	C4—C11—H11A	109.5
C1—C2—H2A	109.9	C4—C11—H11B	109.5
N1—C2—H2B	109.9	H11A—C11—H11B	109.5
C1—C2—H2B	109.9	C4—C11—H11C	109.5
H2A—C2—H2B	108.3	H11A—C11—H11C	109.5
N1—C3—N2	116.6 (2)	H11B—C11—H11C	109.5
N1—C3—S1	123.69 (17)	O2—C12—H12A	109.5
N2—C3—S1	119.71 (15)	O2—C12—H12B	109.5
N3—C4—C11	117.74 (18)	H12A—C12—H12B	109.5
N3—C4—C5	124.46 (19)	O2—C12—H12C	109.5
C11—C4—C5	117.79 (18)	H12A—C12—H12C	109.5
C10—C5—C6	119.0 (2)	H12B—C12—H12C	109.5
C10—C5—C4	120.74 (18)		
			
C3—N2—N3—C4	−178.22 (19)	C10—C5—C6—O2	178.6 (2)
C3—N1—C2—C1	175.6 (3)	C4—C5—C6—O2	−3.4 (3)
C2—N1—C3—N2	171.6 (2)	C10—C5—C6—C7	−2.3 (3)
C2—N1—C3—S1	−8.0 (3)	C4—C5—C6—C7	175.6 (2)
N3—N2—C3—N1	1.3 (3)	O2—C6—C7—C8	179.7 (2)
N3—N2—C3—S1	−179.12 (15)	C5—C6—C7—C8	0.7 (4)
N2—N3—C4—C11	−177.9 (2)	C6—C7—C8—C9	1.8 (4)
N2—N3—C4—C5	0.6 (3)	C7—C8—C9—C10	−2.7 (4)
N3—C4—C5—C10	−87.9 (3)	C6—C5—C10—O1	−179.0 (2)
C11—C4—C5—C10	90.5 (3)	C4—C5—C10—O1	3.0 (3)
N3—C4—C5—C6	94.1 (3)	C6—C5—C10—C9	1.5 (3)
C11—C4—C5—C6	−87.4 (3)	C4—C5—C10—C9	−176.5 (2)
C12—O2—C6—C5	−169.3 (2)	C8—C9—C10—O1	−178.5 (2)
C12—O2—C6—C7	11.7 (4)	C8—C9—C10—C5	1.0 (4)

### Hydrogen-bond geometry (Å, º)

**Table:**

*D*—H···*A*	*D*—H	H···*A*	*D*···*A*	*D*—H···*A*
O1—H1···S1^i^	0.82	2.35	3.1655 (19)	175

Symmetry code: (i) *x*, −*y*, *z*+1/2.
